# Supplementary material for: Simultaneously tuning interlayer spacing and termination of MXenes by Lewis-basic halides
Source: Nat Commun. 2022 Nov 8;13:6731. doi: 10.1038/s41467-022-34569-y (PMC9643510; doi:10.1038/s41467-022-34569-y)
Supplement: Supplementary file 1 — Supplementary Information [file 41467_2022_34569_MOESM1_ESM.pdf]

Supplementary Information

Simultaneously tuning interlayer spacing and termination of MXenes by Lewis-basic halides

Tianze Zhang et al.

Supplementary Table 1. The interlayer spacing of multilayer MXene after intercalation.

**Intercalating solution**	**(Multilayer MXene) Interlayer spacing**	**Ref.**
LiF/HCl	13.50-14.00 Å	1
LiF/HCl	12.42 Å	2
$MgCl_2$	14.29 Å	3
NaCl	11.85 Å	3
N-butyllithium	12.38 Å	4
LiOH	12.38 Å	4
LiOH	13.60 Å	5
$SnCl_4$	12.42 Å	6
LiCl	11.50 Å	6
NaCl	11.01 Å	6
$N_2H_4{\cdot}H_2O$	12.70 Å	7
KOH	12.50 Å	8
HBr/HCl	13.50 Å	9
NaBr, KBr	14.70 Å	This work

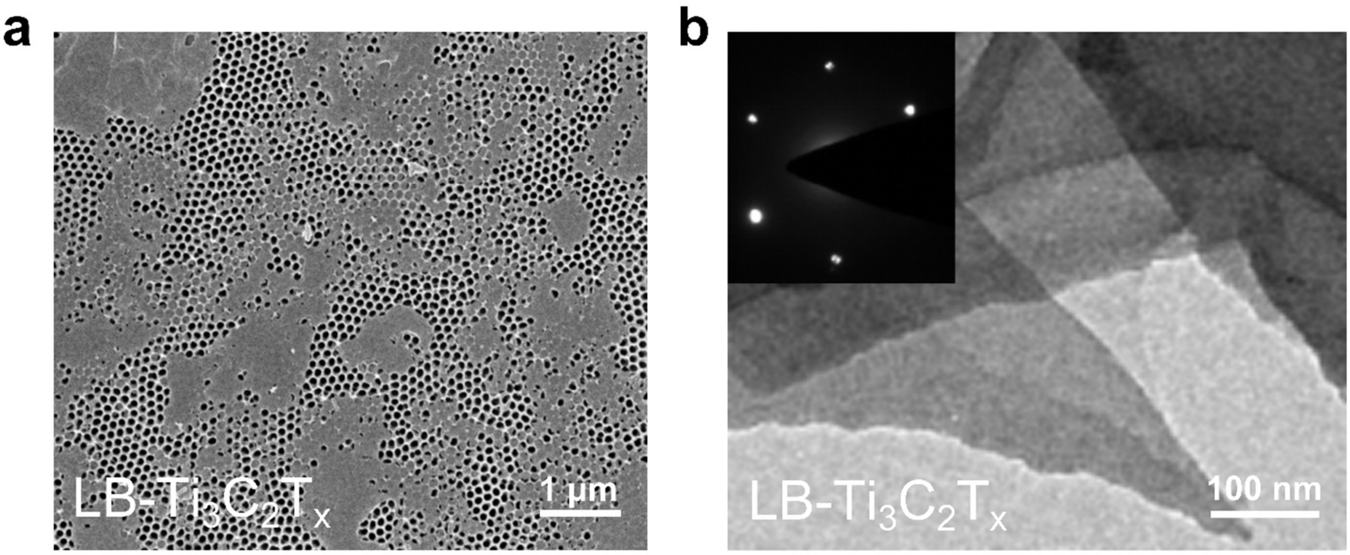

Supplementary Figure 1. Monolayer LB-$Ti_3C_2T_x$ MXene. a-b, SEM and TEM of monolayer LB-$Ti_3C_2T_x$ MXene. SAED shows crystallinity and hexagonal symmetry of the individual flake (Inset of **b**).

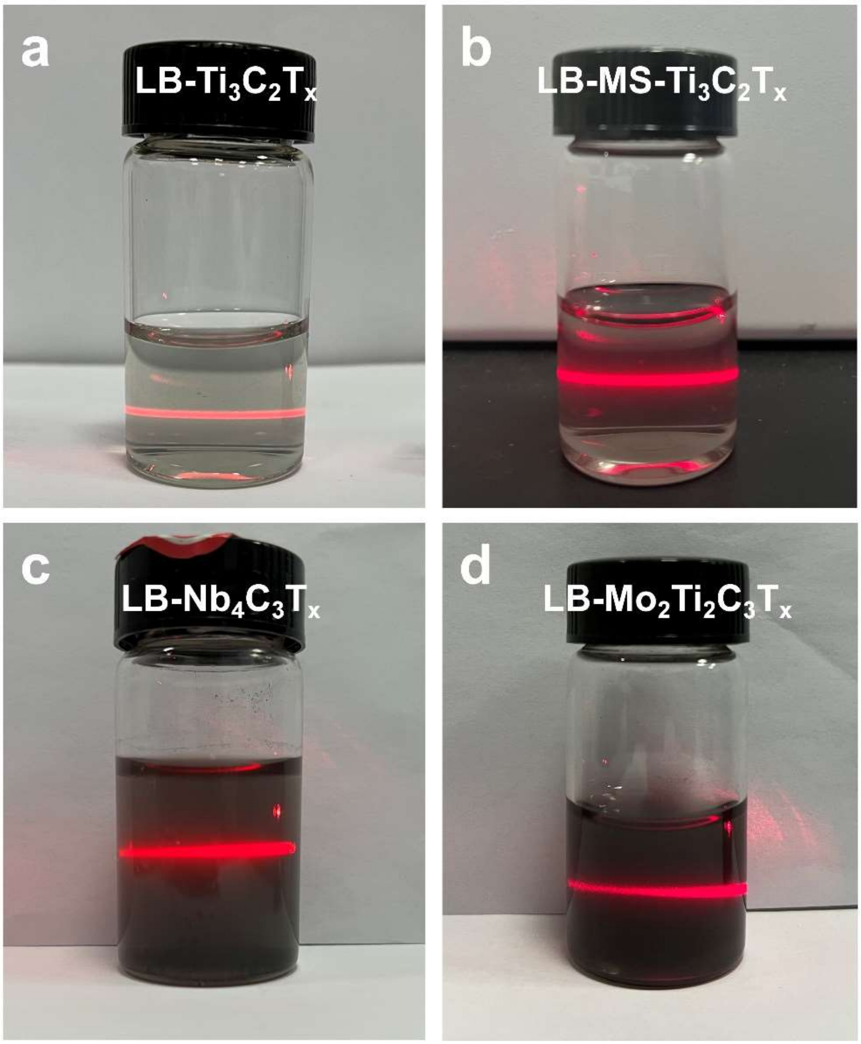

Supplementary Figure 2. Stable colloidal solutions. a-d, Photographs of stable colloidal solutions of LB-$Ti_3C_2T_x$, LB-MS-$Ti_3C_2T_x$, LB-$Nb_4C_3T_x$ and LB-$Mo_2Ti_2C_3T_x$ in water, respectively.

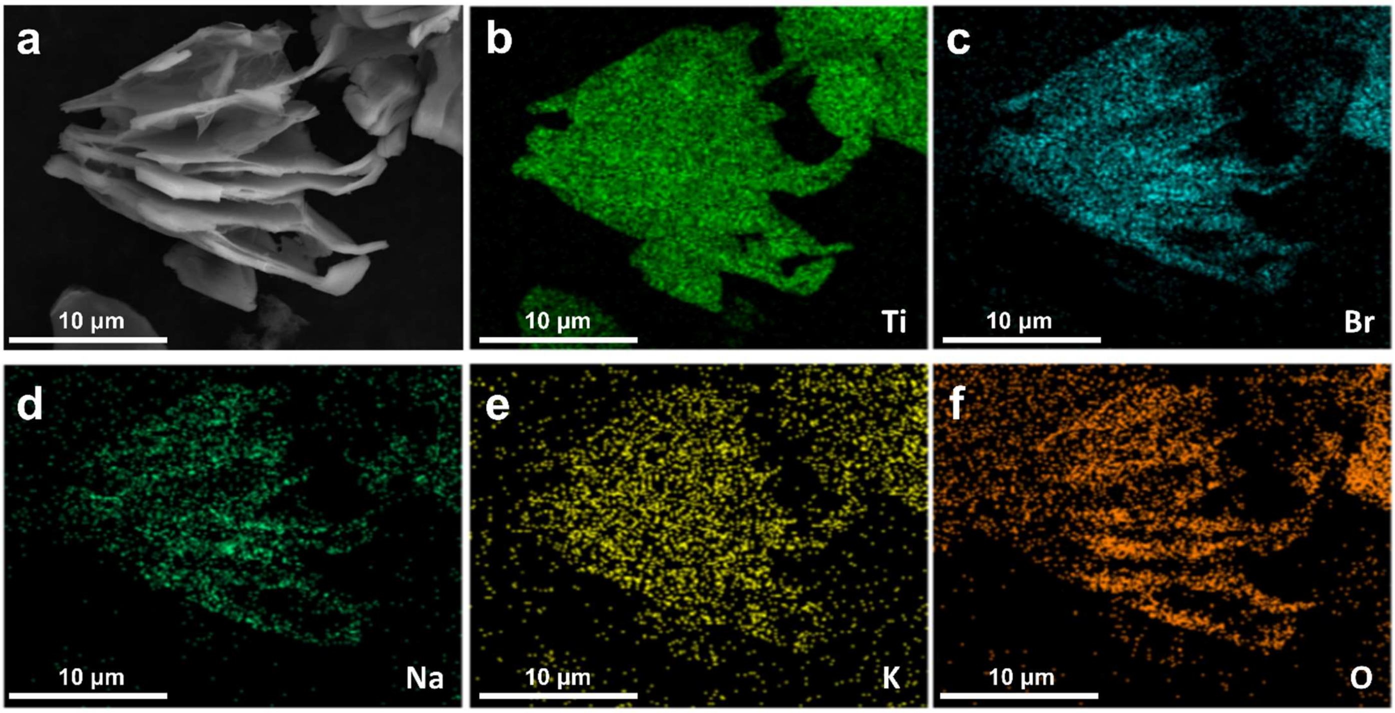

Supplementary Figure 3. a, SEM image and **b-f**, elemental mapping images of Ti, Br, Na, K and O elements of LB-$Ti_3C_2T_x$, respectively.

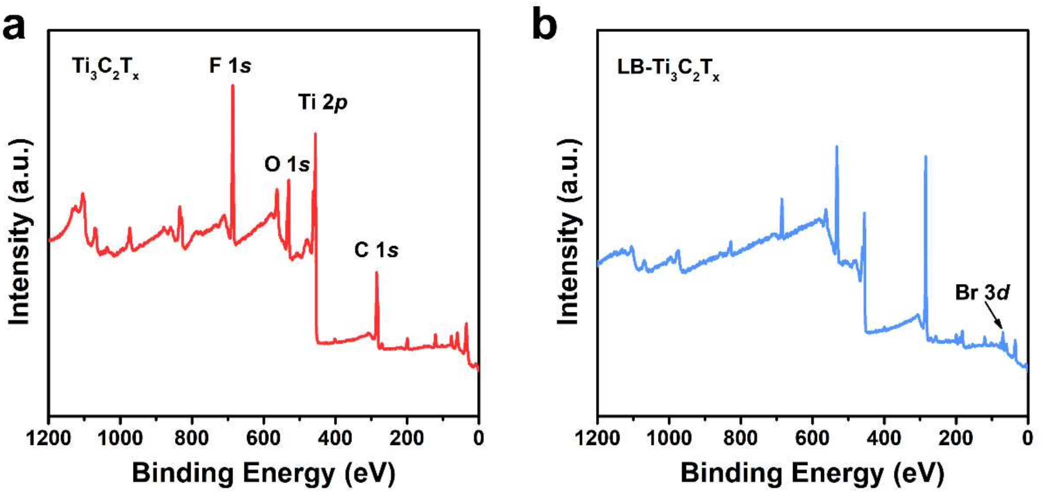

Supplementary Figure 4. XPS spectra of $Ti_3C_2T_x$ (a) and LB-$Ti_3C_2T_x$ (b).

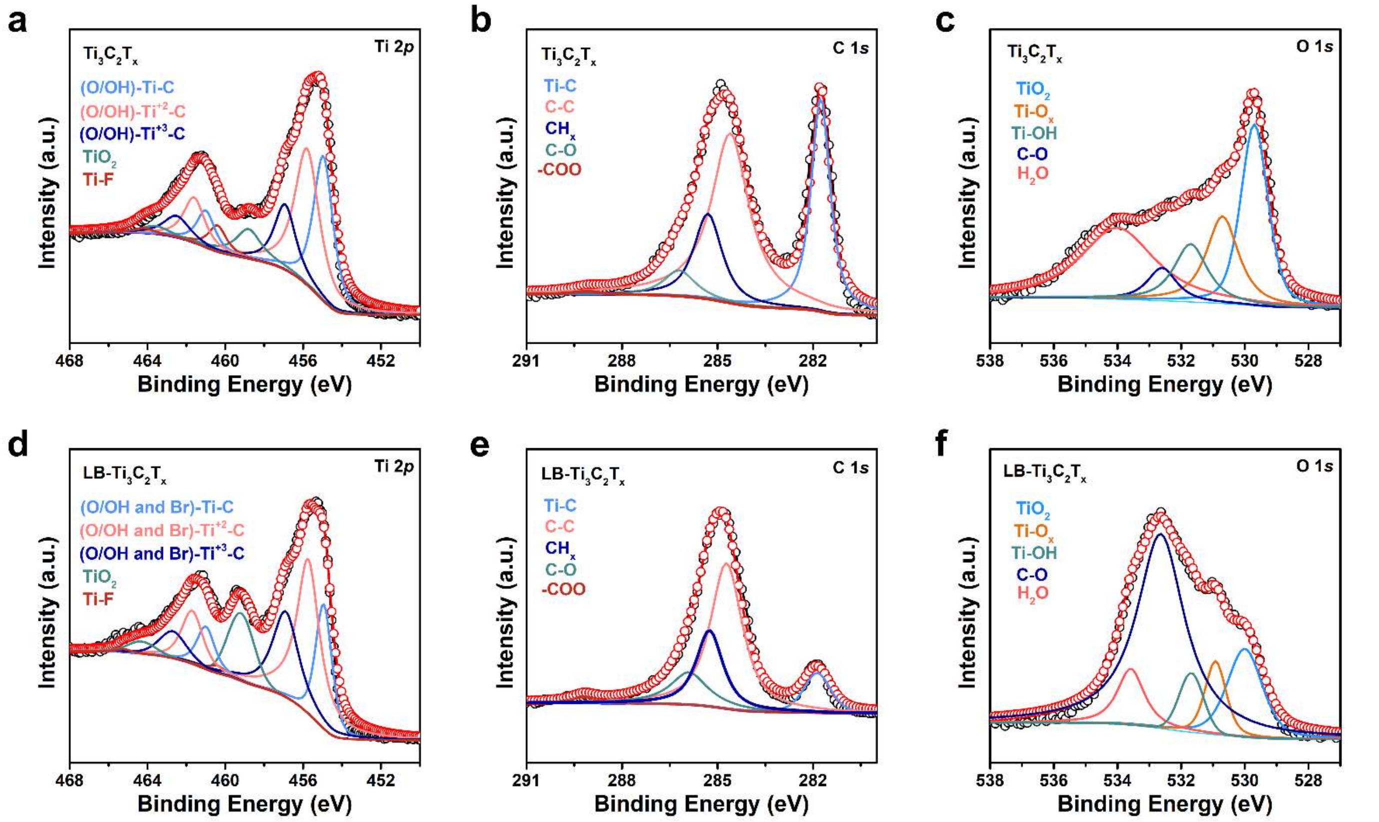

Supplementary Figure 5. High resolution XPS spectra of **(a**, **d)** Ti 2*p*, **(b**, **e)** C 1*s* and **(c, f)** O 1*s* of $Ti_3C_2T_x$ and LB-$Ti_3C_2T_x$.

In the high resolution XPS spectra of $Ti_3C_2T_x$ and LB-$Ti_3C_2T_x$, the Ti 2*p* region (Supplementary Figure 5a and 5d) could be fitted by five components (Detailed peak information is shown in Supplementary Table 2). The largest fractions are attributed to Ti-C bond, and smaller fractions of TiO_2 is also found. After treated by Lewis basic halides, LB-$Ti_3C_2T_x$ exhibits slight increase of TiO_2 and decrease of Ti-F bond, which could be induced by the substitution of -F termination by -Br termination. As shown in Supplementary Figure 5b and 5e, the C 1*s* regions of $Ti_3C_2T_x$ and LB-$Ti_3C_2T_x$ show five peaks, which could be fitted into Ti-C, C-C, CH_x, C-O, and -COO, respectively. The O 1*s* region (Supplementary Figure 5c and 5f) could be assigned to five peaks corresponding to TiO_2, Ti-O_x, Ti-OH, C-O and H_2O, respectively. It's worth noting that the content of C-C and C-O bonds increases in C 1*s* and O 1*s* regions of LB-$Ti_3C_2T_x$. Considering anhydrous diethyl ether and tetrahydrofuran containing C-C and C-O bonds are used for removing salts in our experiment, it is reasonable to speculate that there is a small amount of organic solvent residue on the surface of LB-$Ti_3C_2T_x$ in the process of washing salt.

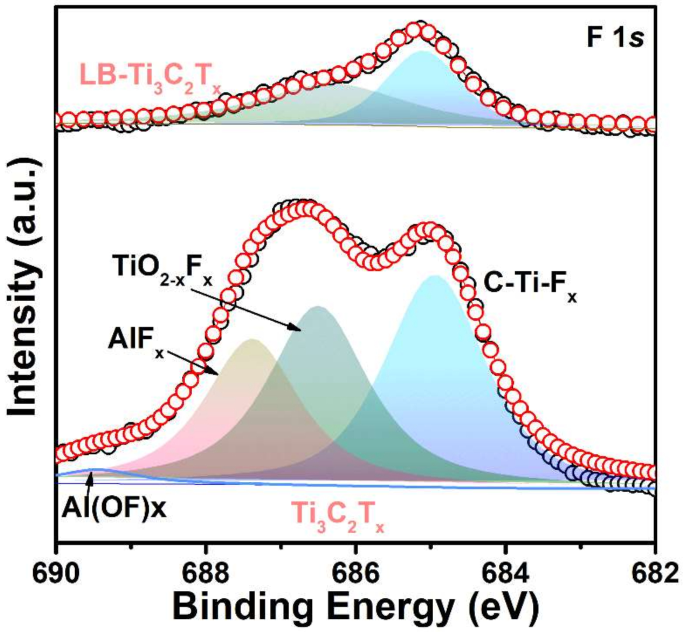

Supplementary Figure 6. High resolution XPS spectra (see Supplementary Table S1 for fitting details) of F 1*s* in $Ti_3C_2T_x$ and LB-$Ti_3C_2T_x$.

The largest component in the F 1*s* region is C-Ti-F_x, which is -F termination. Smaller fractions of $TiO_{2-x}F_x$, AlF_x and $Al(OF)_x$ are also found in the F 1*s* region. The first one is due to surface oxidation, while the latter two are byproducts of the synthesis procedure. After Lewis-basic halides treatment, the peaks of AlF_x and $Al(OF)_x$ disappear, proving AlF_x and $Al(OF)_x$ can be removed. Specifically, the F content in $Ti_3C_2T_x$ is estimated to be 33.94 at.%, whereas the F content in LB-$Ti_3C_2T_x$ is only 6.5 at.%. Moreover, XPS data showed that the content of O changed from 22.1 to 22.6 at.%, proving that Br replaces -F instead of -O termination.

Supplementary Table 2. XPS fitting results of $Ti_3C_2T_x$ and LB-$Ti_3C_2T_x$.

$Ti_3C_2T_x$	BE (eV)	FWHM (eV)	Fraction	Assigned to	Ref.
Ti 2$p_{3/2}$(Ti 2$p_{1/2}$)	455.0 (461.0)	1.20 (1.10)	29.48	(O/OH)-Ti-C	10
	455.8 (461.6)	1.50 (1.30)	38.42	(O/OH)-Ti^{+2}-C	10
	456.9 (462.5)	1.32 (1.60)	17.61	(O/OH)-Ti^{+3}-C	10
	458.8 (463.6)	1.60 (1.70)	9.34	TiO_2	10
	460.3 (464.2)	1.10 (1.20)	5.15	Ti-F	10
C 1*s*	281.8	0.71	28.50	Ti-C	11
	284.8	1.43	48.56	C-C	11
	285.3	1.05	16.76	CH_x	11
	286.2	1.16	5.59	C-O	11
	289.1	1.00	0.59	-COO	11
O 1*s*	529.7	1.01	27.88	TiO_2	11
	530.7	1.20	18.43	Ti-O_x	11
	531.7	1.25	12.76	Ti-OH	11
	532.6	1.09	6.32	C-O	11
	534.0	2.74	34.61	H_2O	11

Supplementary Table 2 continued.

**LB-$Ti_3C_2T_x$**	**BE (eV)**	**FWHM (eV)**	**Fraction**	**Assigned to**	**Ref.**
Ti $2p_{3/2}$(Ti $2p_{1/2}$)	454.9 (461.1)	1.00 (1.10)	20.73	(O/OH and Br)-Ti-C	10
	455.7 (461.7)	1.40 (1.30)	40.58	(O/OH and Br)-Ti^{+2}-C	10
	456.9 (462.7)	1.60 (1.65)	24.07	(O/OH and Br)-Ti^{+3}-C	10
	459.2 (464.3)	1.60 (1.70)	13.24	TiO_2	10
	460.4 (465.2)	1.00 (0.80)	1.38	Ti-F	10
C 1*s*	281.9	1.00	10.10	Ti-C	11
	284.8	1.20	51.44	C-C	11
	285.2	1.00	22.41	CH_x	11
	285.9	1.40	14.30	C-O	11
	289.2	0.92	1.75	-COO	11
O 1*s*	530.0	1.30	16.17	TiO_2	11
	530.9	0.78	8.09	Ti-O_x	11
	531.7	0.90	6.46	Ti-OH	11
	532.6	1.80	60.10	C-O	11
	533.6	1.00	9.18	H_2O	11
Br $3d_{5/2}$(Br $3d_{3/2}$)	68.9 (70.2)	1.56 (1.56)	100.00	Ti-Br	12

BE: Binding Energy

The chemical formula of LB-$Ti_3C_2T_x$ is $Ti_3C_{1.6}O_{0.5}(OH)_{0.4}F_{0.8}Br_{0.6}$ based on XPS.

Supplementary Table 3. Substance species of molten salt system.

**Formula (mol%)**	**Substance species**
$AlBr_3$=100	$AlBr_3$
$AlBr_3$:NaBr:KBr=79.9:5.9:14.2	$AlBr_3$, $[AlBr_4]^-$, $[Al_2Br_7]^-$, Na^+, K^+, **Br^- (Extremely trace)**
$AlBr_3$:NaBr:KBr=60.1:11.7:28.2	$AlBr_3$, $[AlBr_4]^-$, $[Al_2Br_7]^-$, Na^+, K^+, **Br^- (Extremely trace)**
$AlBr_3$:NaBr:KBr=48.7:15.2:36.1	**$AlBr_3$ (Extremely trace)**, $[AlBr_4]^-$, $[Al_2Br_7]^-$, Na^+, K^+, Br^-

A rational set of reactions links these ions to the molecular species.

$$2AlBr_3 \rightleftharpoons Al_2Br_6$$

$$AlBr_3 + Br^- \rightleftharpoons [AlBr_4]^-$$

$$Al_2Br_6 + Br^- \rightleftharpoons [Al_2Br_7]^-$$

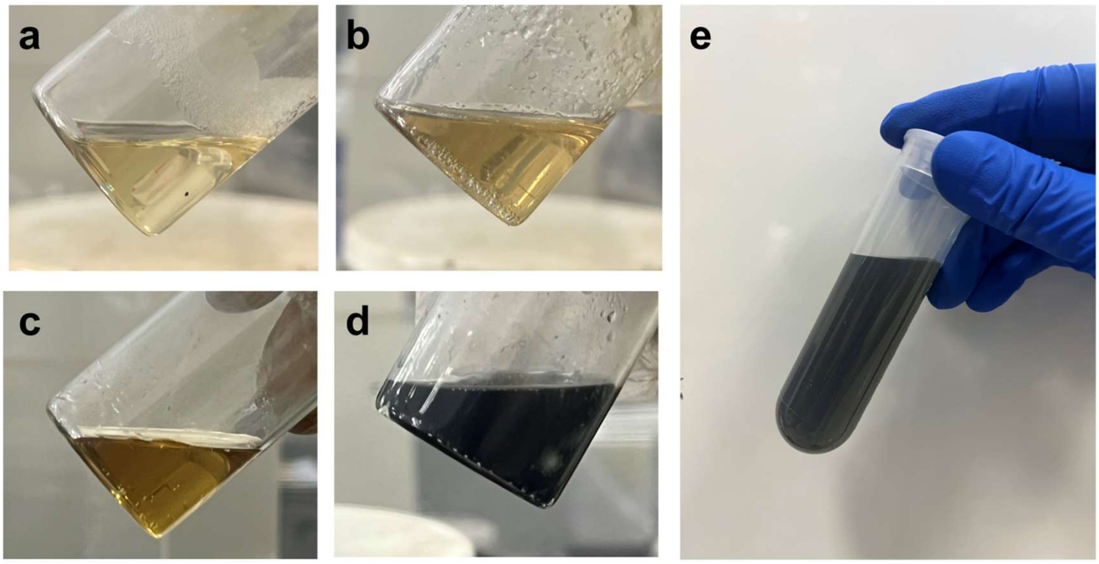

Supplementary Figure 7. Photographs of the $AlBr_3$/NaBr/KBr molten salt and $Ti_3C_2T_x$ in $AlBr_3$/NaBr/KBr molten salt. a-c, Photographs of the $AlBr_3$/NaBr/KBr molten salt with different proportions of $AlBr_3$ (100 mol%, 79.9 mol%, 48.7 mol%). **d**, Photograph of $Ti_3C_2T_x$ in $AlBr_3$/NaBr/KBr molten salt ($AlBr_3$ 48.7%). **e**, Photograph of LB-$Ti_3C_2T_x$ after centrifugation at 3500 rpm for 10 min.

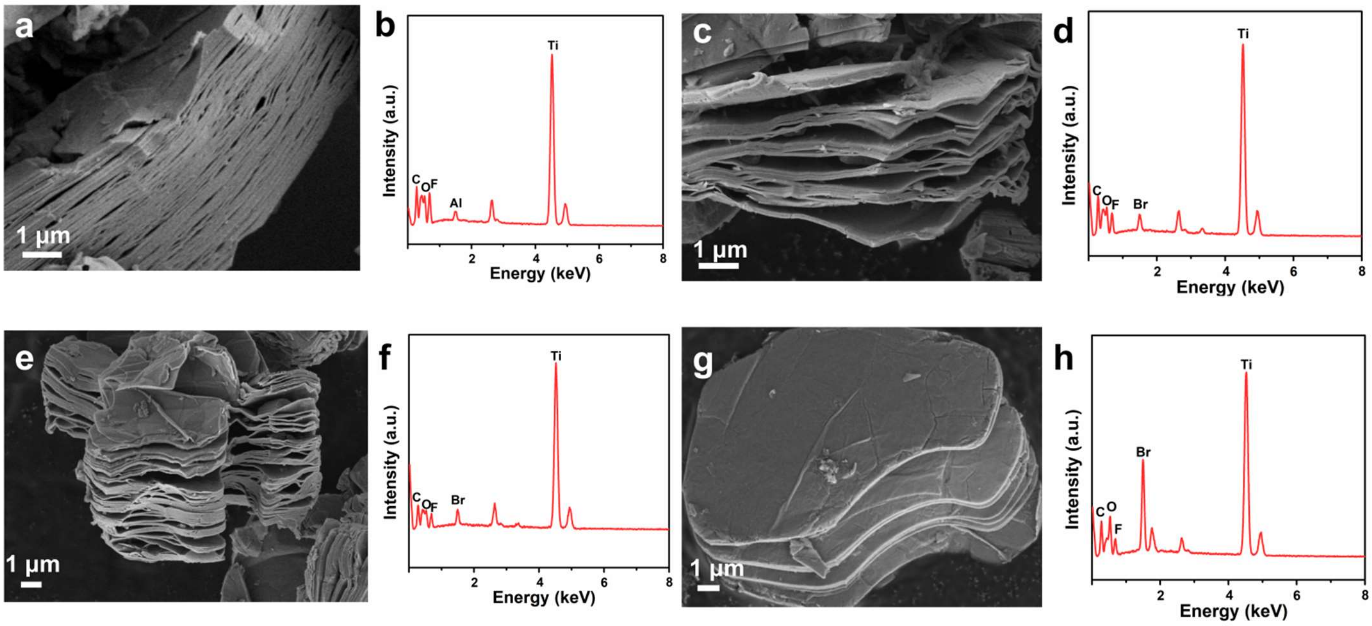

Supplementary Figure 8. SEM and EDS analysis of $Ti_3C_2T_x$ treated by $AlBr_3$/NaBr/KBr molten salts with different component ratios. a-b, SEM image and EDS analysis of $Ti_3C_2T_x$ (100 mol% $AlBr_3$). **c-d**, SEM image and EDS analysis of $Ti_3C_2T_x$ (79.9 mol% $AlBr_3$). **e-f**, SEM image and EDS analysis of $Ti_3C_2T_x$ (60.1 mol% $AlBr_3$). **g-h**, SEM image and EDS analysis of LB-$Ti_3C_2T_x$ (48.7 mol% $AlBr_3$).

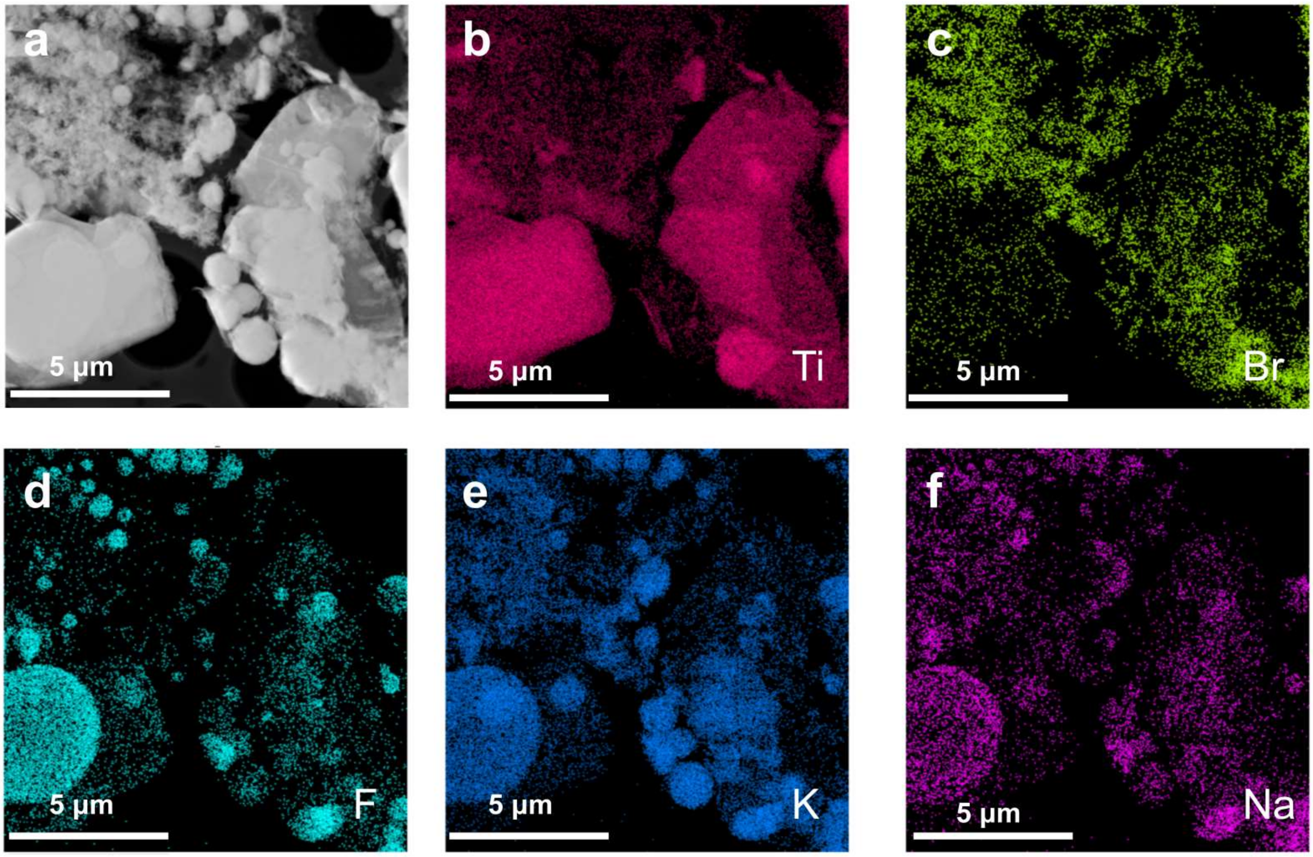

Supplementary Figure 9. a, SEM image and **(b-f)** elemental mapping images of Ti, Br, F, K and Na elements of LB-$Ti_3C_2T_x$, respectively.

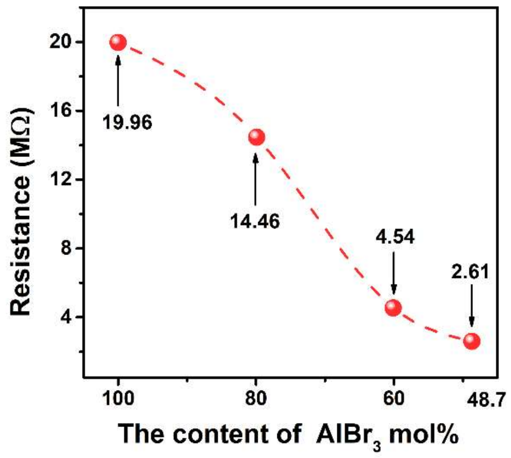

Supplementary Figure 10. Resistance of the $AlBr_3$/NaBr/KBr molten salt with different proportions of $AlBr_3$ (100 mol%, 79.9 mol%, 60.1 mol%, 48.7 mol%).

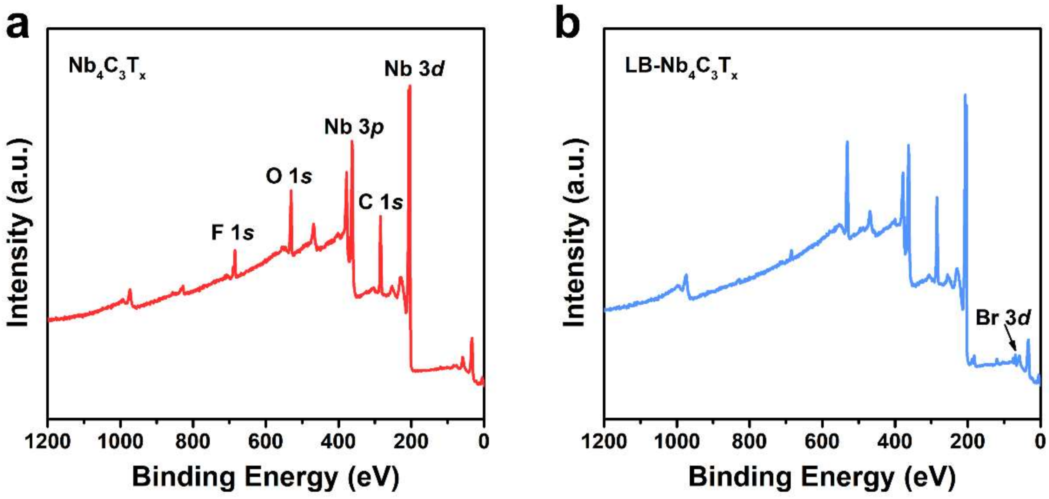

Supplementary Figure 11. XPS spectra of $Nb_4C_3T_x$ and LB-$Nb_4C_3T_x$.

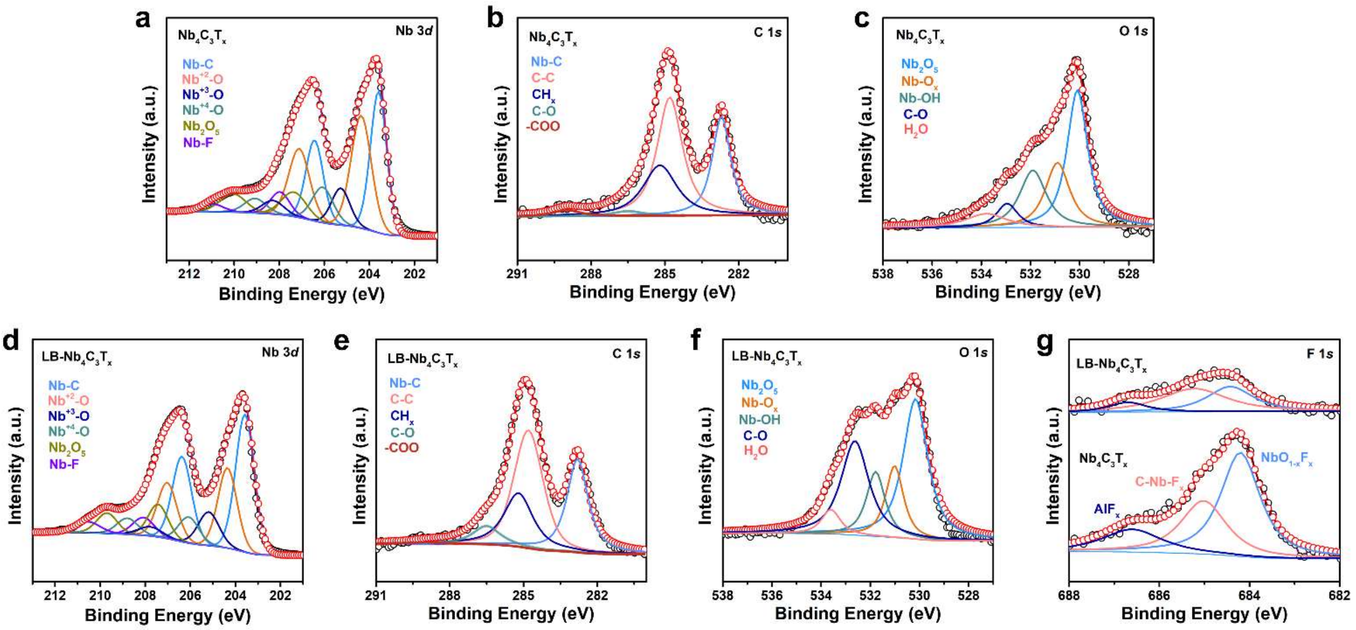

Supplementary Figure 12. High resolution XPS spectra of **(a, d)** Nb 3*d*, **(b, e)** C 1*s*, **(c, f)** O 1*s* and **(g)** F 1*s* of $Nb_4C_3T_x$ and LB-$Nb_4C_3T_x$.

In the high resolution XPS spectra of $Nb_4C_3T_x$ and LB-$Nb_4C_3T_x$, the Nb 3*d* region (Supplementary Figure 12a and 12d) could be fitted by six components (Detailed peak information is shown in Supplementary Table 4), corresponding to Nb-C, Nb^{+2}-O, Nb^{+3}-O, Nb^{+4}-O, Nb_2O_5 and Nb-F, respectively. Supplementary Figure 12b and 12e display the C 1*s* regions of $Nb_4C_3T_x$ and LB-$Nb_4C_3T_x$ that are deconvoluted into five peaks, corresponding to Nb-C, C-C, CH_x, C-O, and -COO, respectively. Supplementary Figure 12c and 12f display the O 1*s* regions which are deconvoluted into five peaks, corresponding to Nb_2O_5, Nb-O_x, Nb-OH, C-O and H_2O, respectively. Similar to the phenomenon of LB-$Ti_3C_2T_x$, the content of C-C and C-O bonds increases. In addition, the -F termination is further confirmed by C-Nb-F_x bond in F 1*s* region (Supplementary Figure 12g). After Lewis basic halides treatment, the C-Nb-F_x bond decreased due to the substitution of -F termination by -Br termination.

Supplementary Table 4. XPS fitting results of $Nb_4C_3T_x$ and LB-$Nb_4C_3T_x$.

$Nb_4C_3T_x$	BE (eV)	FWHM (eV)	Fraction	Assigned to	Ref.
Nb 3$d_{5/2}$(Nb 3$d_{3/2}$)	203.6 (206.4)	0.89 (0.93)	33.40	Nb-C	13
	204.4 (207.1)	1.10 (1.14)	32.96	Nb^{+2}-O	13
	205.3 (207.9)	1.00 (1.04)	9.79	Nb^{+3}-O	13
	206.1 (209.0)	1.14 (1.18)	10.76	Nb^{+4}-O	13
	207.4 (210.0)	1.35 (1.38)	8.84	Nb_2O_5	13
	208.3 (210.9)	1.18 (1.21)	4.25	Nb-F	13
C 1*s*	282.7	0.90	26.86	C-Nb	14
	284.8	1.22	43.70	C-C	14
	285.2	1.62	24.70	CH_x	14
	286.5	1.5	2.15	C-O	14
	289.0	1.39	2.59	-COO	14
O 1*s*	530.1	1.00	40.57	Nb_2O_5	15
	530.9	1.18	22.45	Nb-O_x	15
	531.9	1.28	21.65	Nb-OH	15
	533.0	1.03	7.27	C-O	15
	533.8	1.98	8.06	H_2O	15
F 1*s*	684.2	1.04	52.04	C-Nb-F_x	15
	685.0	1.2	30.89	AlF_x	15
	686.6	1.56	17.07	C-F	15

Supplementary Table 4 continued.

LB-$Nb_4C_3T_x$	BE (eV)	FWHM (eV)	Fraction	Assigned to	Ref.
Nb $3d_{5/2}$(Nb $3d_{3/2}$)	203.6 (206.4)	1.02 (1.05)	41.59	Nb-C	13
	204.4 (207.0)	1.03 (1.06)	24.54	Nb^{+2}-O	13
	205.2 (208.1)	1.14 (1.17)	11.32	Nb^{+3}-O	13
	206.1 (208.8)	1.10 (1.13)	8.51	Nb^{+4}-O	13
	207.4 (209.7)	1.08 (1.11)	10.51	Nb_2O_5	13
	207.8 (210.6)	1.30 (1.34)	3.53	Nb-F	13
C 1*s*	282.8	1.03	28.28	C-Nb	14
	284.8	1.40	41.93	C-C	14
	285.3	1.40	22.13	CH_x	14
	286.5	1.32	7.16	C-O	14
	289.0	1.26	0.50	-COO	14
O 1*s*	530.2	1.20	38.44	Nb_2O_5	15
	531.0	0.86	14.14	Nb-O_x	15
	531.8	0.91	13.42	Nb-OH	15
	532.6	1.35	28.96	C-O	15
	533.6	0.90	5.04	H_2O	15
F 1*s*	684.4	1.22	37.74	C-Nb-F_x	15
	685.2	1.75	50.12	AlF_x	15
	686.7	1.01	12.14	C-F	15
Br $3d_{5/2}$(Br $3d_{3/2}$)	68.6 (69.5)	1.37 (1.37)	100.00	Nb-Br	16

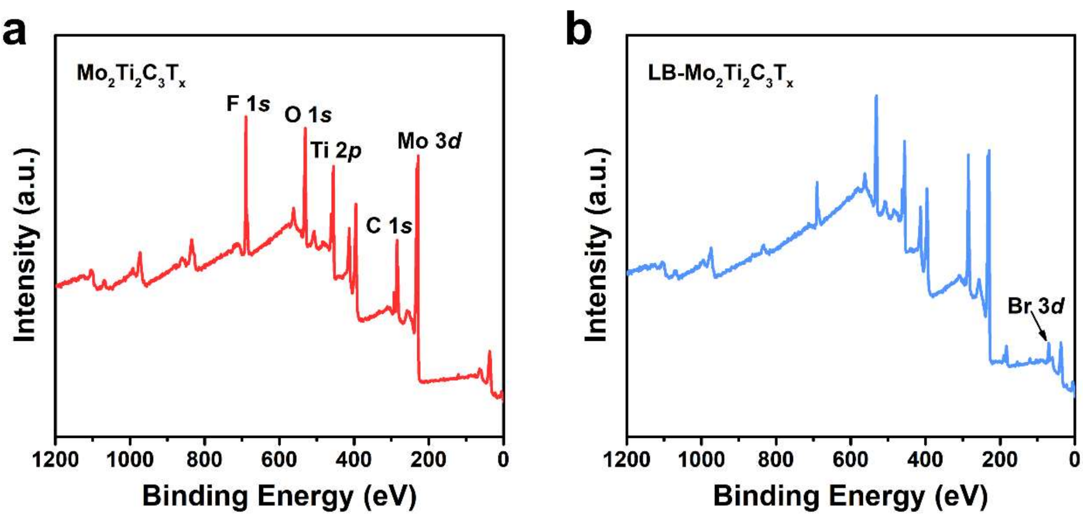

Supplementary Figure 13. XPS spectra of $Mo_2Ti_2C_3T_x$ and LB-$Mo_2Ti_2C_3T_x$.

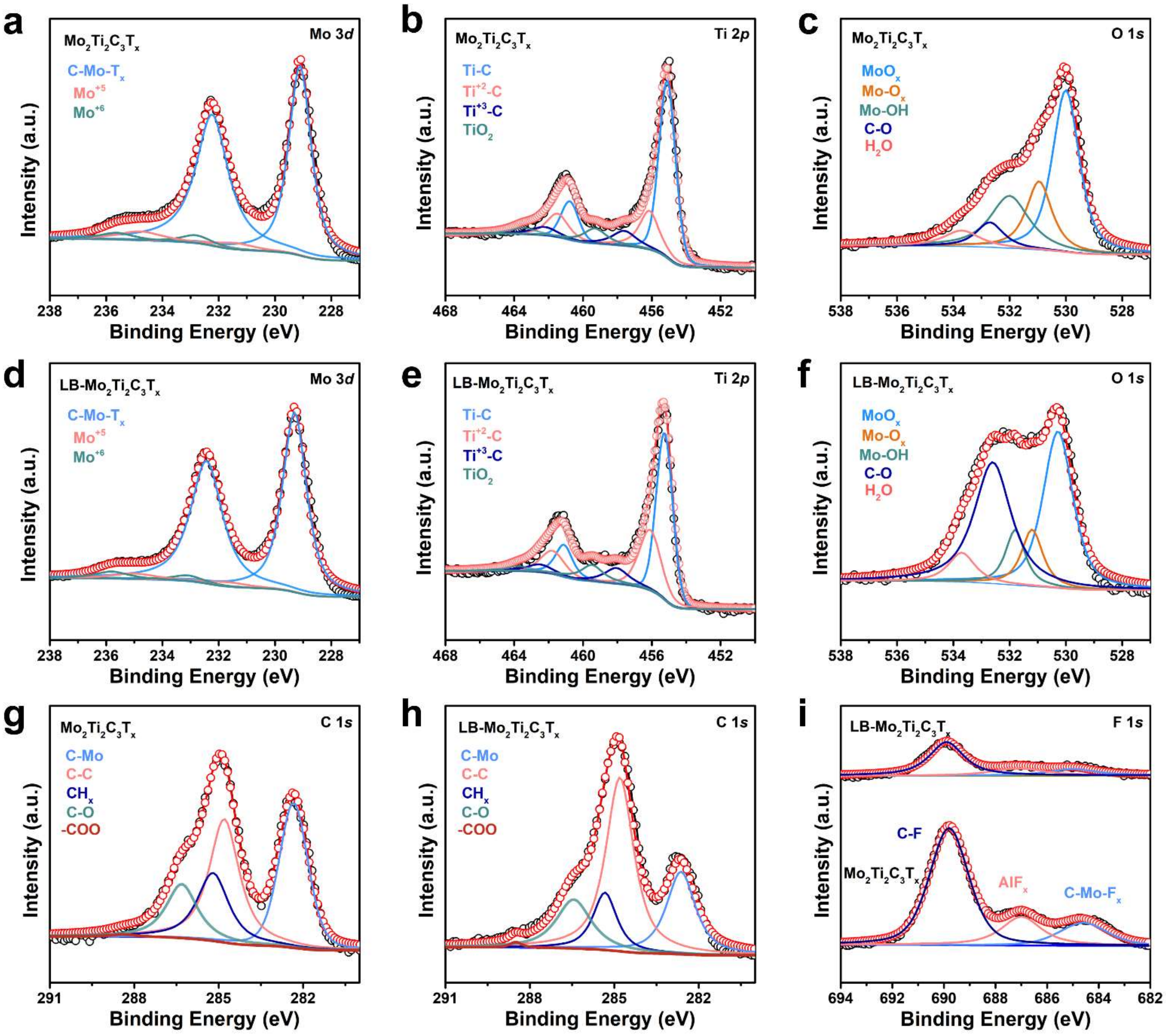

Supplementary Figure 14. High resolution XPS spectra of **(a, d)** Mo 3*d*, **(b, e)** Ti 2*p*, **(c, f)** O 1*s*, **(g, h)** C 1*s* and **(i)** F 1*s* of $Mo_2Ti_2C_3T_x$ and LB-$Mo_2Ti_2C_3T_x$.

In the high resolution XPS spectra of $Mo_2Ti_2C_3T_x$ and LB-$Mo_2Ti_2C_3T_x$, the Mo 3*d* region (Supplementary Figure 14a and 14d) could be fitted by three components (Detailed peak information is shown in Supplementary Table 5). These components are mainly attributed to C-Mo and two small species (Mo^{+5} and Mo^{+6}) belonging to mixed molybdenum oxides. The Ti 2*p* region (Supplementary Figure 14b and 14e) could be fitted by four components, corresponding to Ti-C, Ti^{+2}-C, Ti^{+3}-C and Ti_2O, respectively. Supplementary Figure 14c and 14f display the O 1*s* regions that are deconvoluted into five peaks, corresponding to MoO_x, Mo-O_x, Mo-OH, C-O and H_2O, respectively. The C 1*s* regions (Supplementary Figure 14g and 14h) of $Mo_2Ti_2C_3T_x$ and LB-$Mo_2Ti_2C_3T_x$ show five peaks, which could be fitted into Mo-C, C-C, CH_x, C-O, and -COO, respectively. Similar to the phenomenon of LB-$Ti_3C_2T_x$ and LB-$Nb_4C_3T_x$, the content of C-C and C-O bonds increases. And the F termination is further confirmed by C-Mo-F_x bond in F 1*s* region (Supplementary Figure 14i). After Lewis basic halides treatment, the C-Mo-F_x bond decreased due to the substitution of -F termination by -Br termination.

Supplementary Table 5. XPS fitting results of $Mo_2Ti_2C_3T_x$ and LB-$Mo_2Ti_2C_3T_x$.

$Mo_2Ti_2C_3T_x$	BE (eV)	FWHM (eV)	Fraction	Assigned to	Ref.
Mo $3d_{5/2}$(Mo $3d_{3/2}$)	229.1 (232.2)	0.95 (1.38)	88.40	C-Mo-T_x	17
	231.4 (234.8)	1.70 (1.89)	6.71	Mo^{+5}	17
	232.8 (235.6)	1.30 (1.45)	4.89	Mo^{+6}	17
Ti $2p_{3/2}$(Ti $2p_{1/2}$)	455.1 (460.8)	1.13 (1.20)	60.89	Ti-C	17
	456.1 (461.5)	1.36 (1.50)	20.22	Ti^{+2}-C	17
	457.6 (462.2)	1.70 (1.80)	9.68	Ti^{+3}-C	17
	459.3 (463.5)	1.60 (1.80)	9.21	TiO_2	17
C 1*s*	282.3	1.22	31.31	C-Mo	14
	284.8	1.27	33.73	C-C	14
	285.2	1.33	19.35	CH_x	14
	286.3	1.31	15.12	C-O	14
	288.4	1.00	0.49	-COO	14
O 1*s*	530.0	1.13	44.68	MoO_x	18
	530.9	1.08	19.27	Mo-O_x	18
	532.0	1.63	22.23	Mo-OH	18
	532.7	1.16	7.50	C-O	18
	533.7	1.53	6.32	H_2O	18
F 1*s*	684.6	2.2	15.61	C-Mo-F_x	19
	687.0	1.84	19.10	AlF_x	19
	689.8	1.79	65.29	C-F	19

Supplementary Table 5 continued.

**LB-$Mo_2Ti_2C_3T_x$**	**BE (eV)**	**FWHM (eV)**	**Fraction**	**Assigned to**	**Ref.**
Mo $3d_{5/2}$(Mo $3d_{3/2}$)	229.3 (232.4)	1.01 (1.34)	90.51	C-Mo-T_x	17
	231.6 (235.0)	1.70 (1.89)	4.80	Mo^{+5}	17
	233.1 (235.8)	1.30 (1.45)	4.69	Mo^{+6}	17
Ti $2p_{3/2}$(Ti $2p_{1/2}$)	455.3 (461.1)	1.13 (1.20)	51.80	Ti-C	17
	456.1 (461.8)	1.50 (1.66)	28.39	Ti^{+2}-C	17
	458.0 (462.5)	1.70 (1.80)	9.70	Ti^{+3}-C	17
	459.5 (463.7)	1.60 (1.80)	10.11	TiO_2	17
C 1*s*	282.6	1.22	23.03	C-Mo	14
	284.8	1.18	46.87	C-C	14
	285.3	1.00	13.12	CH_x	14
	286.4	1.46	16.33	C-O	14
	288.5	0.50	0.65	-COO	14
O 1*s*	530.3	1.26	36.64	MoO_x	18
	531.2	0.90	10.18	Mo-O_x	18
	531.8	0.82	9.02	Mo-OH	18
	532.6	1.56	37.64	C-O	18
	533.7	1.12	6.52	H_2O	18
F 1*s*	685.0	2.12	16.82	C-Mo-F_x	19
	687.3	2.50	19.45	AlF_x	19
	689.9	1.59	63.73	C-F	19
Br $3d_{5/2}$(Br $3d_{3/2}$)	69.3 (70.1)	1.68 (1.68)	100.00	Mo-Br	20

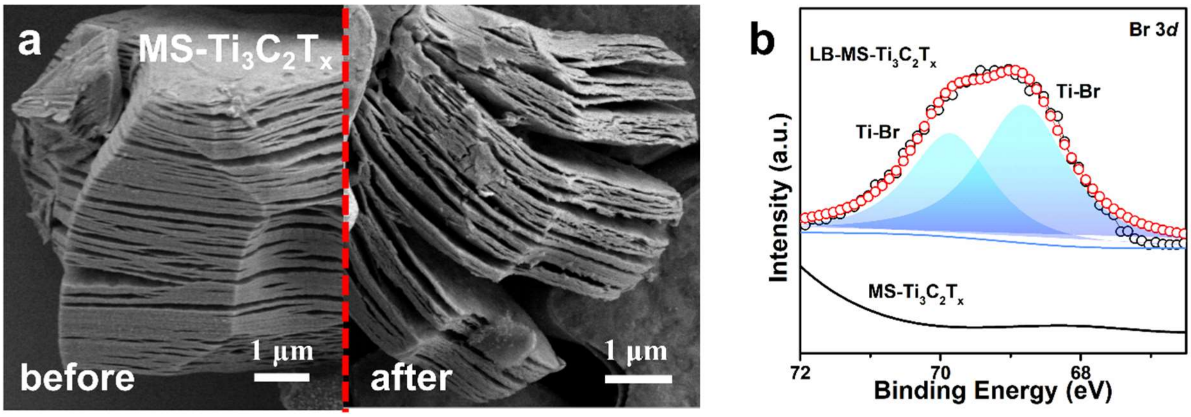

Supplementary Figure 15. a, SEM images of MS-$Ti_3C_2T_x$ MXene before and after Lewis basic halide treatment. **b**, High resolution XPS spectrum of LB-MS-$Ti_3C_2T_x$ MXene.

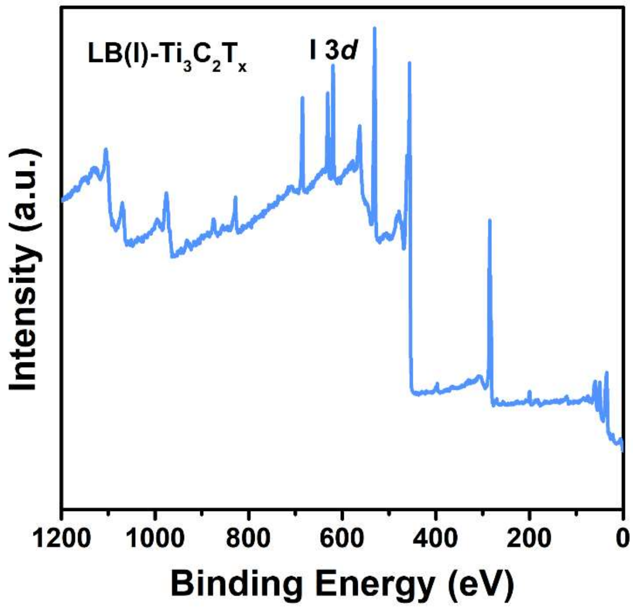

Supplementary Figure 16. XPS spectrum of LB(Iodine)-$Ti_3C_2T_x$.

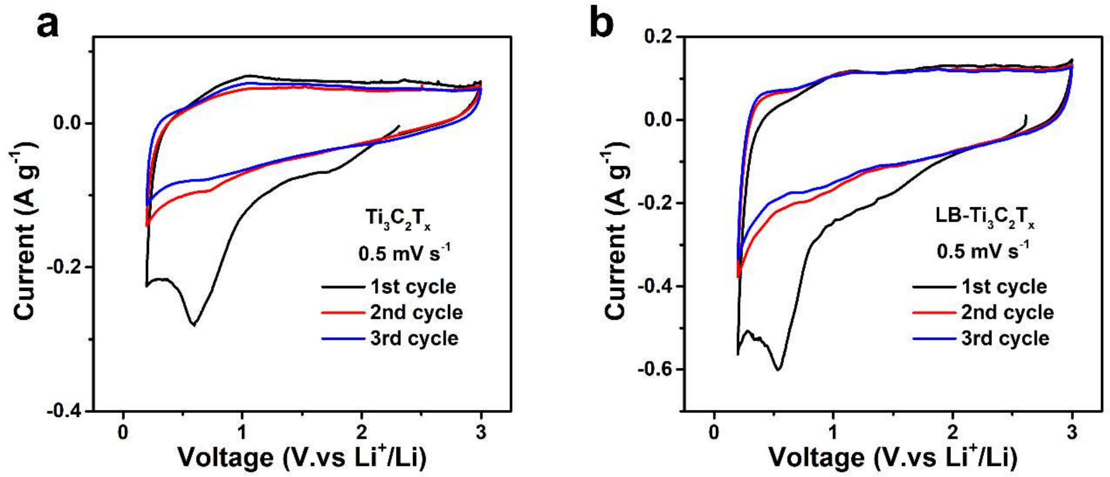

Supplementary Figure 17. First three CV profiles of $Ti_3C_2T_x$ and LB-$Ti_3C_2T_x$ electrodes at 0.5 mV s^{-1}.

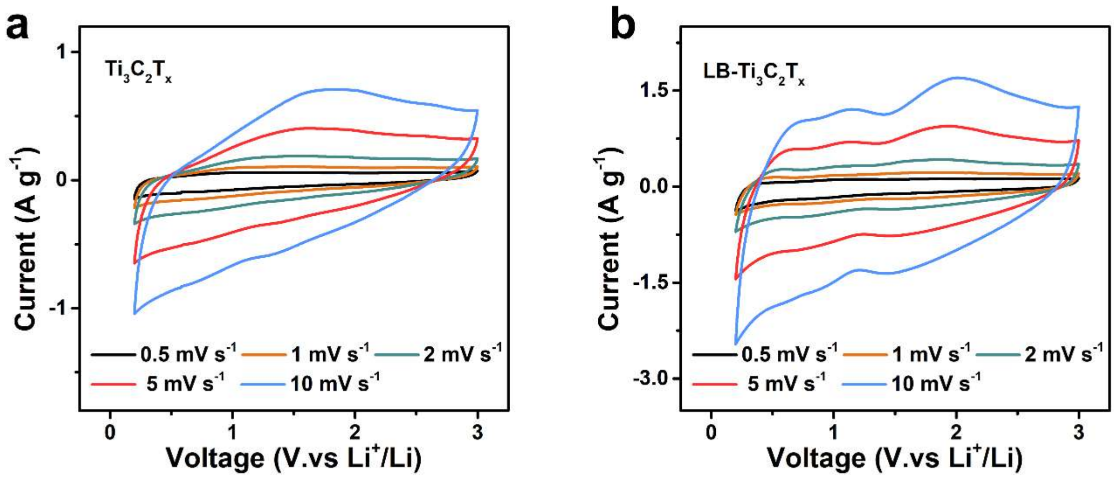

Supplementary Figure 18. CV profiles of $Ti_3C_2T_x$ and LB-$Ti_3C_2T_x$ electrodes at scan rates from 0.5 to 10 mV s^{-1}.

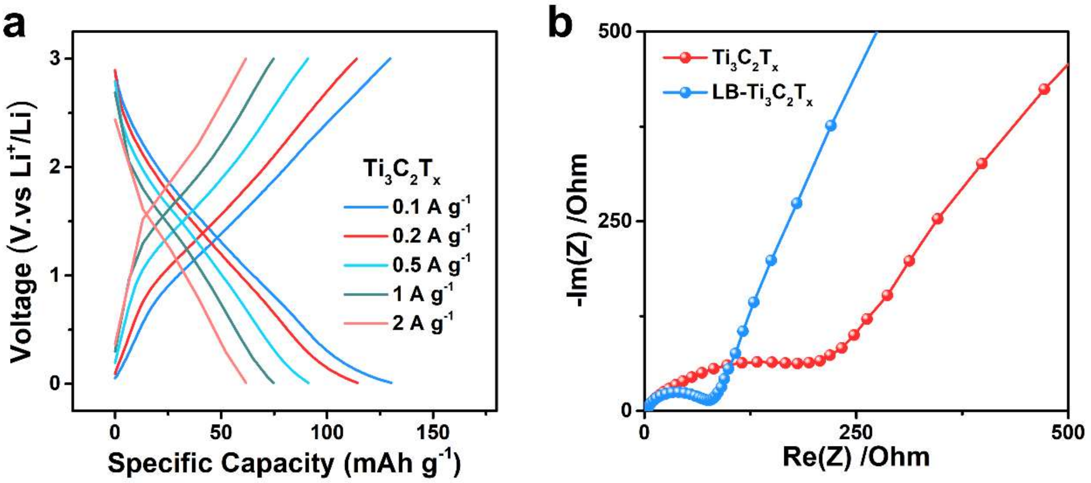

Supplementary Figure 19. **a**, Voltage profiles of $Ti_3C_2T_x$ electrode at various specific currents. **b**, Electrochemical impedance measurements of $Ti_3C_2T_x$ and LB-$Ti_3C_2T_x$ electrodes.

Supplementary Table 6. Li^+ storage capacities of LB-$Ti_3C_2T_x$ electrode at different scan rates.

**Scan rates / mV s^{-1}**	**Capacitance / F g^{-1}**	**Capacity / mAh g^{-1}**	**Time / min (C-rate)**	**Coulomb efficiency / %**
0.5	215	168	0.6	96
1	175	137	1.3	98
2	154	120	2.6	97
5	130	101	6.4	97
10	112	87	13	97
20	94	73	26	97
50	71	55	64	96
100	54	42	128	96

Supplementary Table 7. Li^+ storage capacities of $Ti_3C_2T_x$ electrode at different scan rates.

**Scan rates / mV s^{-1}**	**Capacitance / F g^{-1}**	**Capacity / mAh g^{-1}**	**C-rate**	**Coulomb efficiency / %**
0.5	99	78	0.6	98
1	81	63	1.3	98
2	68	53	2.6	98
5	54	42	6.4	98
10	44	34	13	97
20	35	27	26	97
50	25	19	64	95
100	18	14	128	96

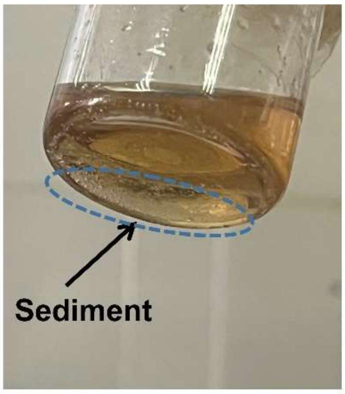

Supplementary Figure 20. Photograph of the $AlBr_3$/NaBr/KBr molten salt with 40.1 mol% of $AlBr_3$.

When the added $AlBr_3$ is less than 48.7 mol% (for instance, 40.1 mol%), sediments appear at the bottom of the solution, indicating the solubility limit (the content of $AlBr_3$ in NaBr/KBr/$AlBr_3$) may be around 48.7 mol%.

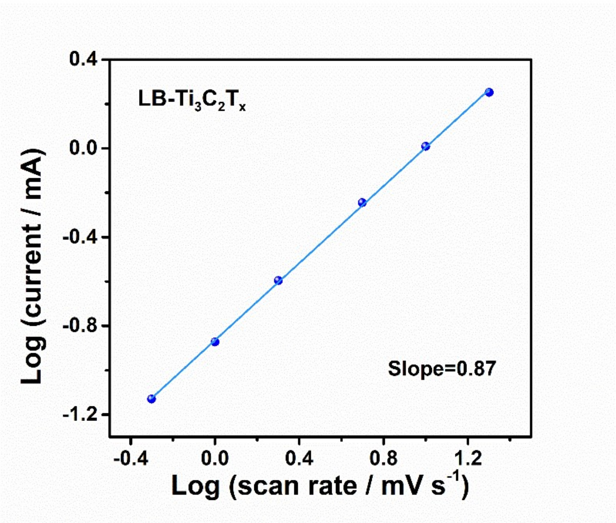

Supplementary Figure 21. Change of the peak current with the potential scan rate in log scale.

According to the power-law relationship $i=av^b$ (*Nat. Mat.* 2013, 12, 518-522), the correlation between the peak current (i) and the sweep rate (v) can be studied to distinguish the storage process of the charge, where a and b denote arbitrary coefficients[21]. When b closes to 0.5, it shows diffusion-dominated charge storage. When b closes to 1.0, it shows capacitance-contributed dominant charge storage. In our work, the calculated b value (0.87) closes to 1, indicating that the LB-$Ti_3C_2T_x$ electrode is dominated by capacitance-contributed charge storage.

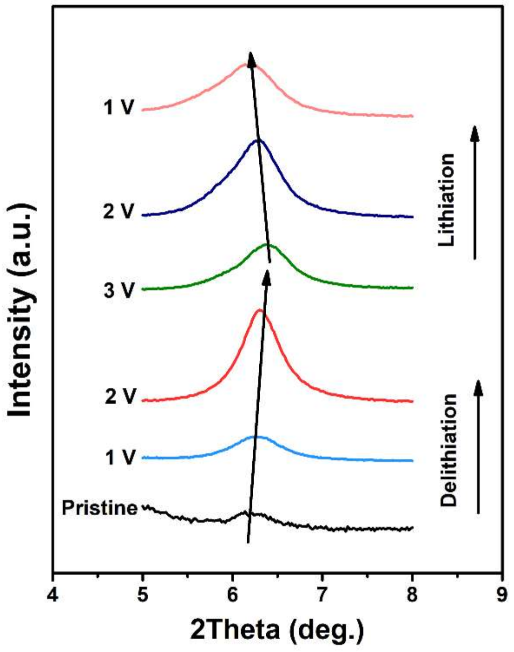

Supplementary Figure 22. Ex-situ XRD patterns of LB-$Ti_3C_2T_x$ electrode at different potential during the charge and discharge processes.

The interlayer spacing decreased from 14.4 (6.13°) to 13.8 Å (6.38°) during lithium deintercalation, and increases from 13.8 (6.38°) back to 14.3 Å (6.17°) during lithium intercalation, which means the interlayer structure of LB-$Ti_3C_2T_x$ is pretty stable during cycling.

Supplementary References

1. Ghidiu, M., Lukatskaya, M. R., Zhao, M. Q., Gogotsi, Y. & Barsoum, M. W. Conductive two-dimensional titanium carbide 'clay' with high volumetric capacitance. *Nature* **516**, 78-81 (2014).

2. Lipatov. A. et al. Effect of synthesis on quality, electronic properties and environmental stability of individual monolayer Ti_3C_2 MXene flakes. *Adv. Electron. Mater.* **2**, 1600255 (2016).

3. Al-Temimy, A. et al. Impact of cation intercalation on the electronic structure of $Ti_3C_2T_x$ MXenes in sulfuric acid. *ACS Appl. Mater. Interfaces* **12**, 15087-15094 (2020).

4. Chen, X. F. et al. N-butyllithium-treated $Ti_3C_2T_x$ MXene with excellent pseudocapacitor performance. *ACS Nano* **13**, 9449-9456 (2019).

5. Wang, H. B. et al. Achieving high-rate capacitance of multi-layer titanium carbide (MXene) by liquid-phase exfoliation through Li-intercalation. *Electrochem. Commun.* **81**, 48-51 (2017).

6. Hu, A. L., Y, J., Zhao, H. Z., Zhang, H,. Li, W. One-step synthesis for cations intercalation of two-dimensional carbide crystal Ti_3C_2 MXene. *Appl. Surf. Sci.* **505**, 144538 (2020).

7. Mashtalir, O. et al. Effect of hydrazine intercalation on structure and capacitance of 2D titanium carbide (MXene). *Nanoscale* **8**, 9128-9133 (2016).

8. Li, J. et al. Achieving high pseudocapacitance of 2D titanium carbide (MXene) by cation intercalation and su rface modification. *Adv. Energy Mater.* **7**, 1602725 (2017).

9. Anion adsorption, $Ti_3C_2T_z$ MXene multilayers, and their effect on claylike swelling. *J. Phys. Chem. C* **122**, 23172−23179 (2018).

10. Natu. V. et al. A critical analysis of the X-ray photoelectron spectra of $Ti_3C_2T_z$ MXenes. *Matter* **4**, 1224–1251 (2021).

11. Kamysbayev, V. et al. Covalent surface modifications and superconductivity of two-dimensional metal carbide MXenes. *Science* **369**, 979-983 (2020).

12. Jawaid, A. et al. Halogen etch of Ti_3AlC_2 MAX phase for MXene fabrication. *ACS Nano* **15**, 2771-2777 (2021).

13. Zhao, S. S. et al. Li-ion uptake and increase in interlayer spacing of Nb_4C_3 MXene. E*nergy Stor. Mater*. **8**, 42-48 (2017).

14. Jayaweera, P. M., Quah, E. L. & Idriss, H. Photoreaction of ethanol on TiO_2 (110) single-crystal surface. *J. Phys. Chem. C* **111**, 1764-1769 (2007).

15. Yang, J. et al. Two-dimensional Nb-based M_4C_3 solid solutions (MXenes). J. Am. Ceram. Soc. **99**, 660-666 (2016).

16. Maya. L. Ammonolysis of niobium(V) bromide. *Inorg. Chem.* **26**, 1459-1462 (1987).

17. Gandla, D., Zhang, F. M. & Tan, D. Q. Advantage of larger interlayer spacing of a $Mo_2Ti_2C_3$ MXene free-standing film electrode toward an excellent performance supercapacitor in a binary ionic liquid-organic electrolyte. *ACS Omega* **7**, 7190-7198 (2022).

18. Yamamoto, S. et al. In situ x-ray photoelectron spectroscopy studies of water on metals and oxides at ambient conditions. *J. Phys.: Condens. Matter* **20**, 184025 (2008).

19. Halim, J. et al. Synthesis and Characterization of 2D Molybdenum Carbide (MXene). *Adv. Funct. Mater*. **26**, 3118–3127 (2016).

20. Kumar, P. et al. Hexamolybdenum clusters supported on graphene oxide: Visible-light induced photocatalytic reduction of carbon dioxide into methanol. *Carbon* **94**, 91-100 (2015).

21. Augustyn, V. et al. High-rate electrochemical energy storage through Li^+ intercalation pseudocapacitance. *Nat. Mater.* **12**, 518–522 (2013).
