# Supplementary material for: Simultaneously tuning interlayer spacing and termination of MXenes by Lewis-basic halides
Source: Nat Commun. 2022 Nov 8;13:6731. doi: 10.1038/s41467-022-34569-y (PMC9643510; doi:10.1038/s41467-022-34569-y)
Supplement: Supplementary file 3 — Reporting Summary [file 41467_2022_34569_MOESM3_ESM.pdf]

nature portfolio

Corresponding author(s): Xu Xiao

Last updated by author(s): Oct 22, 2022

Reporting Summary

Nature Portfolio wishes to improve the reproducibility of the work that we publish. This form provides structure for consistency and transparency in reporting. For further information on Nature Portfolio policies, see our Editorial Policies and the Editorial Policy Checklist.

Statistics

For all statistical analyses, confirm that the following items are present in the figure legend, table legend, main text, or Methods section.

n/a	Confirmed	
☐	☒	The exact sample size (*n*) for each experimental group/condition, given as a discrete number and unit of measurement
☐	☒	A statement on whether measurements were taken from distinct samples or whether the same sample was measured repeatedly
☐	☒	The statistical test(s) used AND whether they are one- or two-sided<br>*Only common tests should be described solely by name; describe more complex techniques in the Methods section.*
☐	☒	A description of all covariates tested
☐	☒	A description of any assumptions or corrections, such as tests of normality and adjustment for multiple comparisons
☐	☒	A full description of the statistical parameters including central tendency (e.g. means) or other basic estimates (e.g. regression coefficient) AND variation (e.g. standard deviation) or associated estimates of uncertainty (e.g. confidence intervals)
☐	☒	For null hypothesis testing, the test statistic (e.g. F, t, r) with confidence intervals, effect sizes, degrees of freedom and P value noted<br>*Give P values as exact values whenever suitable.*
☐	☒	For Bayesian analysis, information on the choice of priors and Markov chain Monte Carlo settings
☐	☒	For hierarchical and complex designs, identification of the appropriate level for tests and full reporting of outcomes
☐	☒	Estimates of effect sizes (e.g. Cohen's *d*, Pearson's *r*), indicating how they were calculated

Our web collection on statistics for biologists contains articles on many of the points above.

Software and code

Policy information about availability of computer code

Data collection	Not applicable
Data analysis	Not applicable

For manuscripts utilizing custom algorithms or software that are central to the research but not yet described in published literature, software must be made available to editors and reviewers. We strongly encourage code deposition in a community repository (e.g. GitHub). See the Nature Portfolio guidelines for submitting code & software for further information.

Data

Policy information about availability of data

All manuscripts must include a data availability statement. This statement should provide the following information, where applicable:

- Accession codes, unique identifiers, or web links for publicly available datasets
- A description of any restrictions on data availability
- For clinical datasets or third party data, please ensure that the statement adheres to our policy

The datasets generated during and/or analysed during the current study are available from the corresponding author on reasonable request.

Human research participants

Policy information about studies involving human research participants and Sex and Gender in Research.

Reporting on sex and gender	Not applicable
Population characteristics	Not applicable
Recruitment	Not applicable
Ethics oversight	Not applicable

Note that full information on the approval of the study protocol must also be provided in the manuscript.

Field-specific reporting

Please select the one below that is the best fit for your research. If you are not sure, read the appropriate sections before making your selection.

[ ] Life sciences [ ] Behavioural & social sciences [x] Ecological, evolutionary & environmental sciences

For a reference copy of the document with all sections, see nature.com/documents/nr-reporting-summary-flat.pdf

Ecological, evolutionary & environmental sciences study design

All studies must disclose on these points even when the disclosure is negative.

Study description	The surface and interfacial chemistry are of significance on controlling the properties of two-dimensional transition metal carbides and nitrides (MXenes). Numerous efforts have been devoted to the regulation of Ti3C2Tx MXene, however, tuning interlayer spacing and surface halogen termination of other MXenes (besides Ti3C2Tx) is rarely reported while demanded. Here we propose a Lewis-basic halides treatment, which is capable of simultaneously engineering the interlayer spacing and surface termination of various MXenes. Benefited from the abundant desolvated halogen anions and cations in molten state Lewis-basic halides, the -F termination was substituted by nucleophilic reaction and the interlayer spacing was enlarged. Ti3C2Tx MXene treated by this method showed a high specific capacity of 229 mAh g-1 for Li+ storage, which is almost 2 times higher than pristine one. Considering the universality, our method provides a new approach to regulating the properties of MXenes, which may expand their potential applications in energy storage, optoelectronics and beyond.
Research sample	MXene treated by Lewis-basic halides (denoted as LB-MXene)
Sampling strategy	XRD, SEM, STEM, XPS, TEM are used to characterize the materials. The measurements are useful to understand the structure and the component of the materials.
Data collection	The datas are collected by all the authors though the characterization of the instruments.
Timing and spatial scale	0-1200 eV of XPS; 5-10° of XRD;خ خ
Data exclusions	No data exclusions
Reproducibility	The experiments are conducted more than two times and the reproducibility is good.
Randomization	The materials were allocated into groups by the components.
Blinding	Not applicable

Did the study involve field work? [ ] Yes [x] No

Reporting for specific materials, systems and methods

We require information from authors about some types of materials, experimental systems and methods used in many studies. Here, indicate whether each material, system or method listed is relevant to your study. If you are not sure if a list item applies to your research, read the appropriate section before selecting a response.

Materials & experimental systems

n/a	Involved in the study
☒	☐ Antibodies
☒	☐ Eukaryotic cell lines
☒	☐ Palaeontology and archaeology
☒	☐ Animals and other organisms
☒	☐ Clinical data
☒	☐ Dual use research of concern

Methods

n/a	Involved in the study
☒	☐ ChIP-seq
☒	☐ Flow cytometry
☒	☐ MRI-based neuroimaging
